# Melatonin Reduces Excitability in Dorsal Root Ganglia Neurons with Inflection on the Repolarization Phase of the Action Potential

**DOI:** 10.3390/ijms20112611

**Published:** 2019-05-28

**Authors:** Klausen Oliveira-Abreu, Nathalia Maria Silva-dos-Santos, Andrelina Noronha Coelho-de-Souza, Francisco Walber Ferreira-da-Silva, Kerly Shamyra da Silva-Alves, Ana Carolina Cardoso-Teixeira, José Cipolla-Neto, José Henrique Leal-Cardoso

**Affiliations:** 1 Laboratório de Eletrofisiologia, Instituto Superior de Ciências Biomédicas, Universidade Estadual do Ceará, Fortaleza 60714-903, CE, Brazil; klausenoliveira@gmail.com (K.O.-A.); nathaliamsantos14@gmail.com (N.M.S.-d.-S.); andrelinanoronha@gmail.com (A.N.C.-d.-S.); walberferreira@gmail.com (F.W.F.-d.-S.); shamyrabio@gmail.com (K.S.d.S.-A.); anacarolina.acct@gmail.com (A.C.C.-T.); 2 Laboratório de Neurobiologia, Instituto de Ciências Biomédicas 1, Universidade de São Paulo, São Paulo 05508-000, SP, Brazil; cipolla@icb.usp.br

**Keywords:** melatonin, DRG, dorsal root ganglion, excitability, action potential, passive electric properties

## Abstract

Melatonin is a neurohormone produced and secreted at night by pineal gland. Many effects of melatonin have already been described, for example: Activation of potassium channels in the suprachiasmatic nucleus and inhibition of excitability of a sub-population of neurons of the dorsal root ganglia (DRG). The DRG is described as a structure with several neuronal populations. One classification, based on the repolarizing phase of the action potential (AP), divides DRG neurons into two types: Without (N_0_) and with (N_inf_) inflection on the repolarization phase of the action potential. We have previously demonstrated that melatonin inhibits excitability in N_0_ neurons, and in the present work, we aimed to investigate the melatonin effects on the other neurons (N_inf_) of the DRG neuronal population. This investigation was done using sharp microelectrode technique in the current clamp mode. Melatonin (0.01–1000.0 nM) showed inhibitory activity on neuronal excitability, which can be observed by the blockade of the AP and by the increase in rheobase. However, we observed that, while some neurons were sensitive to melatonin effect on excitability (excitability melatonin sensitive—EMS), other neurons were not sensitive to melatonin effect on excitability (excitability melatonin not sensitive—EMNS). Concerning the passive electrophysiological properties of the neurons, melatonin caused a hyperpolarization of the resting membrane potential in both cell types. Regarding the input resistance (R_in_), melatonin did not change this parameter in the EMS cells, but increased its values in the EMNS cells. Melatonin also altered several AP parameters in EMS cells, the most conspicuously changed was the (dV/dt)_max_ of AP depolarization, which is in coherence with melatonin effects on excitability. Otherwise, in EMNS cells, melatonin (0.1–1000.0 nM) induced no alteration of (dV/dt)_max_ of AP depolarization. Thus, taking these data together, and the data of previous publication on melatonin effect on N_0_ neurons shows that this substance has a greater pharmacological potency on N_inf_ neurons. We suggest that melatonin has important physiological function related to N_inf_ neurons and this is likely to bear a potential relevant therapeutic use, since N_inf_ neurons are related to nociception.

## 1. Introduction

Melatonin (*N*-acetyl-5-methoxytryptamine) is a neurohormone produced and secreted at night by the pineal gland. It was first isolated by Lerner and colleagues in 1958 [1]. Ever since, melatonin has been studied in several tissues and conditions [2]. Many effects of melatonin have already been described, for example: Activation of potassium ion channels in the suprachiasmatic nucleus (SCN) [3], and inhibition of excitability of trigeminal ganglion neurons that participate in nociception [4].

In several countries, melatonin is sold over the counter by pharmacies and drugstores as a nutrient supplement [5], and has been therapeutically used for treatment of sleep disorders [6]. Amongst the biological effects of melatonin, its antioxidant activity is to be mentioned. This is one of the best characterized properties of melatonin that is potentiated by its ability to stimulate the transcription and activity of the antioxidant enzymatic systems. These activities and properties not only show the importance of melatonin as a hormone and its relevance as a potential therapeutic agent, but are also the main reasons to support the suggestions of melatonin to treat neurodegenerative diseases, for example: Diabetes mellitus [7], Parkinson [8], and Alzheimer’s disease [9,10].

Due to its amphiphilicity, melatonin can cross the cell and organelle membranes, and act directly intracellularly or bind to its membrane receptors, MT_1_ and MT_2_, to exert its effects. There are several papers reporting the expression of these receptors in central nervous system and in peripheral organs (for review, see [2]). Concerning neural functions, there are several reports connecting melatonin and pain [11,12].

Several works already described the dorsal root ganglia (DRG) as a structure with several neuronal populations [13,14]. In a previous work from our research group we classified the complete population of DRG neurons into two types, based on having (N_inf_) or not (N_0_) inflection on the descending (repolarizing) phase of the action potential (AP), clearly and easily identifiable in the first derivative dV/dt of the voltage signal [14]. Using this classification we previously investigated the effects of melatonin on the neuronal excitability of N_0_ cells [15]. The remaining neurons, N_inf_, concerning melatonin effects that have not yet been studied, are generally suggested to correspond to nociceptors [13,14,16,17,18] and are reported to have different types of functions, as compared to N_0_ cells [13,14]. Because of that, the present study aimed to investigate the effects of melatonin on the N_inf_ neuronal population.

## 2. Results

### 2.1. Neuronal Sample Characterization

In this present study we investigated the effects of melatonin on neuronal excitability of N_inf_ cells. A total of 109 cells were used. The control values of electrophysiological parameters of the 109 N_inf_ neurons used in this investigation can be found in Table 1. For comparison, the control parameters obtained from N_0_ neurons, previously published [15], were reproduced on Table 1. The following parameters of N_inf_ statistically differed (*p* < 0.05, ANOVA, followed by Holm-Sidak test) from N_0_ cells: input resistance (R_in_), amplitude, duration, maximum ascendant inclination and maximum descendant inclination of action potential.

### 2.2. Melatonin Effect on the Excitability of N_inf_ DRG Neurons

**Figure 1:**
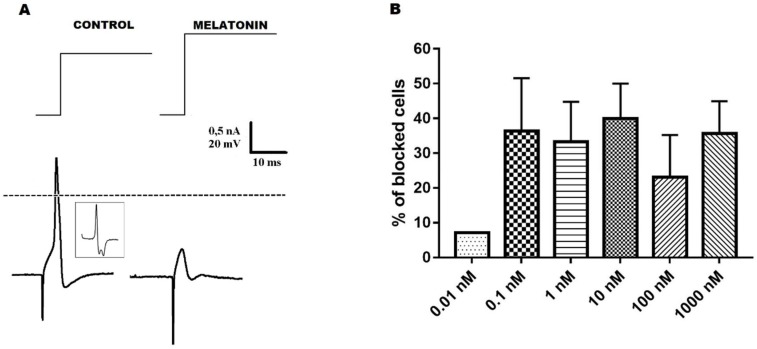
Melatonin-induced AP blockade in N_inf_ neurons. Panel **A** shows representative traces illustrating melatonin effect on the AP of N_inf_ neurons. Traces represent: upper row–current pulses; lower row–left, control AP; right–exposure to melatonin (1000.0 nM). Insert–d*V*/d*t* of control AP. Broken line is zero voltage reference. Panel **B** shows the percentage of neurons with AP triggering blocked (ordinate) by melatonin 0.01, 0.1, 1.0, 10.0, 100.0, and 1000.0 nM (abscissa). These data, expressed in percentage values, are presented as means ± SEM.

Melatonin (0.01–1000.0 nM) showed inhibitory activity on neuronal excitability of N_inf_ cells by blocking the AP triggering (Figure 1, panel A). Melatonin 0.01, 0.1, 1.0, 10.0, 100.0, and 1000.0 nM induced, respectively, blockade on 1 out of 14 cells (1/14; 7.1%), (4/11; 36.3%), 6/18 (33.3%); 10/25 (40.0%); 3/13 (23.0%), and 10/28 (35.7%) cells (Figure 1, panel B).

**Figure 2:**
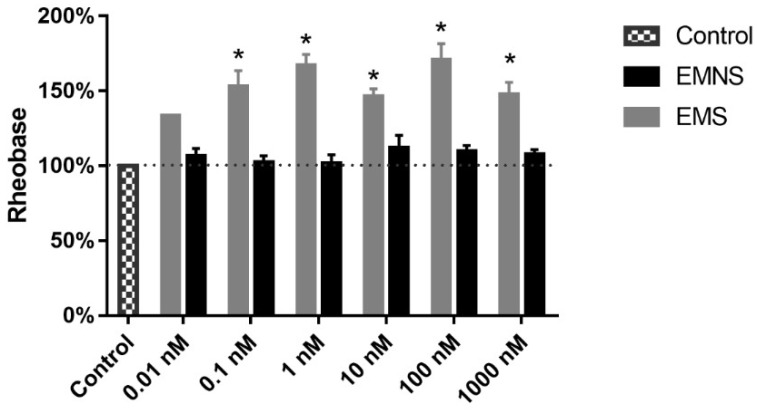
Effect of melatonin on the rheobase of N_inf_ excitability melatonin sensitive (EMS) and excitability melatonin not sensitive (EMNS) cells. These data, expressed in normalized values, are presented as means ± SEM and rheobase absolute control values are found in Table 1. The number of experiments of N_inf_ EMS neurons at concentrations of 0.01, 0.1, 1.0, 10.0, 100.0, and 1000.0 nM were 1, 4, 6, 10, 3, and, 10, respectively. The number of experiments of N_inf_ EMNS neurons at concentrations of 0.01, 0,1, 1.0, 10.0, 100.0, and 1000.0 nM were 13, 7, 12, 15, 10, and 18, respectively. Ordinate, percentage of own control value. Abscissa, melatonin concentration (control, only vehicle). The symbol * indicates statistical difference compared to control (*p* < 0.05, paired Student’s *t*-test).

The rheobase of the neurons were measured; it was significantly altered on the cells in which the AP blockade occurred, but not on those neurons without blockade (*p* < 0.05, paired Student’s *t*- test), in all tested concentrations (Figure 2). These experiments, therefore, showed that regarding melatonin effects on N_inf_ neuronal excitability, there are cells on which melatonin blocked the AP and others in which it did not, regardless of the melatonin concentration, it is to say, these cells are classifiable as neurons sensitive to melatonin effect on excitability (excitability melatonin sensitive—EMS), or neurons not sensitive to melatonin effect on excitability (excitability melatonin not sensitive—EMNS), respectively. Because of that we investigated whether similar type of effects of melatonin (0.01–1000.0 nM) would occur on other neuronal electrophysiological parameters not so essentially related to excitability.

### 2.3. Melatonin Effect on Passive Properties of N_inf_ DRG Neurons

**Figure 3:**
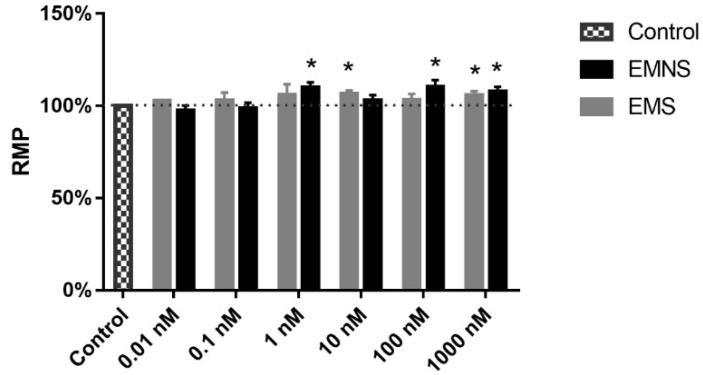
Effect of melatonin on the resting membrane potential (RMP) of N_inf_ excitability melatonin sensitive (EMS) and excitability melatonin not sensitive (EMNS) cells. These data, expressed in normalized values, are presented as means ± SEM and RMP absolute control values are found in Table 1. The number of experiments of N_inf_ EMS neurons at concentrations of 0.01, 0.1, 1.0, 10.0, 100.0, and 1000.0 nM were 1, 4, 6, 10, 3, and, 10, respectively. The number of experiments of N_inf_ EMNS neurons at concentrations of 0.01, 0.1, 1.0, 10.0, 100.0, and 1000.0 nM were 13, 7, 12, 15, 10, and 18, respectively. The symbol * indicates statistically significant difference as compared to control (*p* < 0.05, paired Student’s *t*-test).

We measured two passive properties of the neurons in control situation and in presence of melatonin. Concerning resting membrane potential (RMP), on both EMS and EMNS cells, melatonin showed a tendency to produce RMP hyperpolarization. This tendency did not seem to be quantitatively different from EMS to EMNS cells; in both types of neurons, at several concentrations, the hyperpolarizing RMP alteration (Figure 3) was statistically significant (*p* < 0.05, paired Student’s *t*-test). However, no special type of concentration-hyperpolarization relationship was detected.

**Figure 4:**
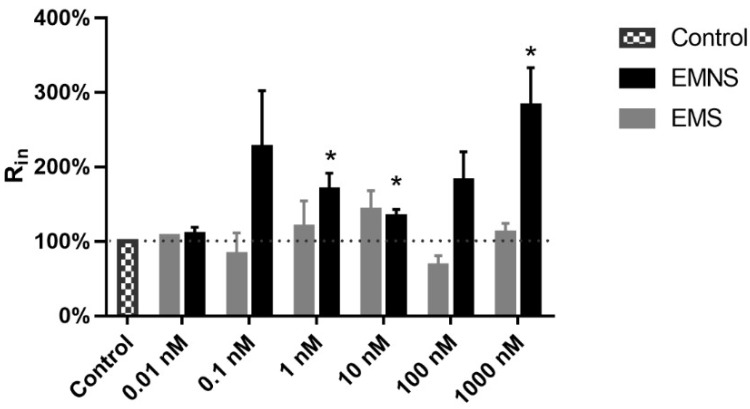
Effect of melatonin on the input resistance (R_in_) of N_inf_ excitability melatonin sensitive (EMS) and excitability melatonin not sensitive (EMNS) cells. These data, expressed in normalized values, are presented as means ± SEM and R_in_ absolute control values are found in Table 1. The number of experiments of N_inf_ EMS neurons at concentrations of 0.01, 0.1, 1.0, 10.0, 100.0, and 1000.0 nM were 1, 4, 6, 10, 3, and, 10, respectively. The number of experiments of N_inf_ EMNS neurons at concentrations of 0.01, 0.1, 1.0, 10.0, 100.0, and 1000.0 nM were 13, 7, 12, 15, 10, and 18, respectively. The symbol * indicates statistically significant difference as compared to control (*p* < 0.05, paired Student’s *t*-test).

Concerning the input resistance (R_in_), on EMS cells, melatonin did not change this parameter (Figure 4). On EMNS cells (Figure 4), there was a conspicuous increase in R_in_, which was significant (*p* < 0.05, paired Student’s *t*-test) at concentration 1.0, 10.0, and 1000.0 nM.

### 2.4. Melatonin Effect on Active Properties of N_inf_ Cells

**Figure 5:**
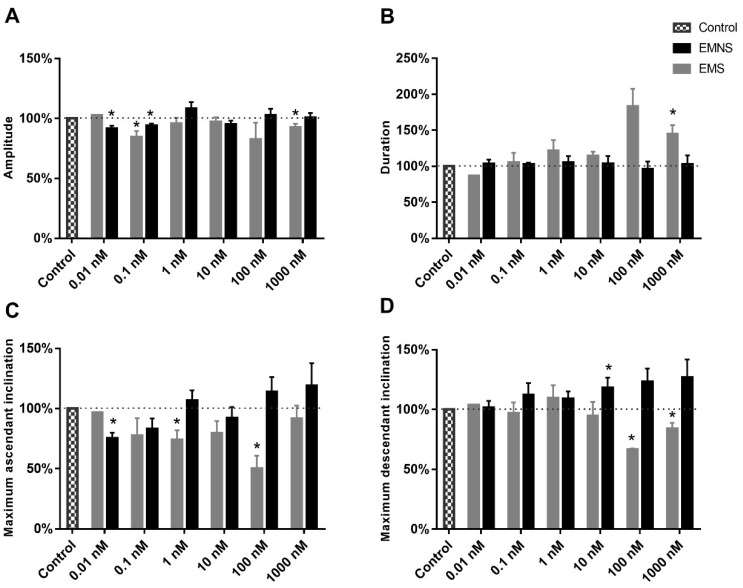
Effect of melatonin on the amplitude (**A**), duration (**B**), maximum ascendant inclination (**C**), and maximum descendant inclination (**D**) of N_inf_ excitability melatonin sensitive (EMS) and excitability melatonin not sensitive (EMNS) cells. These data, expressed in normalized values, are presented as means ± SEM and absolute control values for these parameters are found in Table 1. The number of experiments of N_inf_ EMS neurons at concentrations of 0.01, 0.1, 1.0, 10.0, 100.0, and 1000.0 nM were 1, 4, 6, 10, 3, and, 10, respectively. The number of experiments of N_inf_ EMNS neurons at concentrations of 0.01, 0.1, 1.0, 10.0, 100.0, and 1000.0 nM were 13, 7, 12, 15, 10, and 18, respectively. The symbol * indicates statistically significant difference as compared to control (*p* < 0.05, paired Student’s *t*-test).

In relation to the action potential amplitude on EMS cells, a small magnitude decrease in this parameter, in the presence of melatonin, was observed (Figure 5A); this decrease reached significance at 0.1 and 1000.0 nM (*p* < 0.05, paired Student’s t-test). Concerning AP amplitude on EMNS cells, a tendency was more clearly for no alteration of this parameter, but a significant decrease was observed for the 0.01 and 0.1 nM of melatonin (Figure 5A).

In relation to the AP duration on EMS cells, a tendency for an increase in this parameter, in the presence of the greater concentrations of melatonin, was observed (Figure 5B), which was significant at 1000.0 nM. Regarding AP duration, on EMNS cells, there was no alteration (Figure 5B).

Concerning the (dV/dt)_max_ of the ascending phase of the AP, on EMS cells, a tendency to decrease in this parameter in presence of melatonin was observed (Figure 5C), which was significant at 1.0 and 100.0 nM. For the (dV/dt)_max_ of the ascending phase of the AP, on EMNS cells, there was no significant alteration (Figure 5C), except for 0.01 nM melatonin, in which case a decrease of this parameter was observed.

Regarding the (dV/dt)_max_ of the descending phase of the AP, on EMS cells, in presence of melatonin, a significant decrease in this parameter at 100.0 and 1000.0 nM was observed (Figure 5D). For the (dV/dt)_max_ of the descending phase of the AP, on EMNS cells, there was significant increase at 10.0 nM (Figure 5D).

### 2.5. Melatonin Effect on Na^+^/K^+^-ATPase Activity

**Figure 6:**
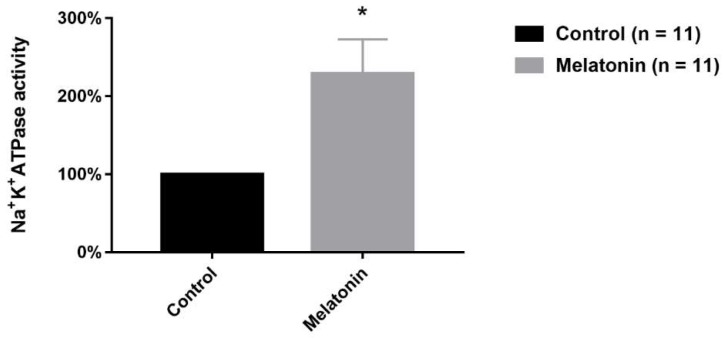
Effect of melatonin (100.0 nM) on Na^+^K^+^-ATPase activity. Activities expressed as paired measurements of individual increases related to individual controls normalized as percentage of control activity (means ± SEM). Na^+^K^+^-ATPase activity is expressed as nmol (Pi)/µg protein/hour. The symbol * indicates statistically significant difference as compared to control (*p* < 0.05, paired Student’s *t*-test).

Since melatonin promoted hyperpolarization in N_inf_ and N_0_ neurons, we investigated whether this substance alters Na^+^/K^+^-ATPase activity. Our results show that melatonin increases this activity (Figure 6). In control, and upon 15 min of exposure of the preparation to melatonin (100.0 nM), the average Na^+^/K^+^-ATPase activity was 17.5 ± 3.81, and 28.5 ± 4.41 nmol (Pi)/µg protein/hour (*n* =11), respectively. When we measured this melatonin-induced intensification of activity, expressed as individual increases related to individual control (paired measurements) as a percentage of control activity, the increase amounted to 228.9 ± 44.16 % (*n* = 11) (Figure 6).

## 3. Discussion

The main finding of this work is that melatonin reduces the excitability of N_inf_ cells, a sub population of the DRG neurons. This activity on the excitability was observed from concentration ≥0.1 nM melatonin, suggesting a hormonal-type effect. Additionally, two characteristics on this effect deserve to be emphasized: 1 - melatonin induces this effect with a conspicuously greater pharmacological potency as compared to the similar, previously reported effect [15] on N_0_ DRG neurons and, 2 – the pharmacological efficacy of this effect, clearly saturates at 0.1 nM. This saturation at 0.1 nM melatonin demonstrates that concerning melatonin sensitivity, there are two clearly distinct N_inf_ population, one with excitability sensitive to this substance and another not sensitive. Based on our knowledge, and on what is available in the international literature, this is the first work to demonstrate the electrophysiological effects of melatonin on excitability of N_inf_ cells and the difference of its effects on different types of DRG neurons of rats.

We initially verified whether the difference between N_0_ and N_inf_ cells included electrophysiological parameters other than the presence of the inflection on the repolarizing phase of the AP. In order to achieve that, we compared the control values of N_inf_ cells obtained in the present study with the control values of N_0_ cells previously obtained [15]. We found that the following electrophysiological parameters were statistically different from N_inf_ to N_0_: R_in_, amplitude, duration, maximum ascendant inclination, and maximum descendant inclination of the AP (Table 1). It was only regarding the rheobase and RMP that N_inf_ and N_0_ did not differ. These differences strongly suggest that, in fact, we worked with another neuronal population in the present study. Furthermore, we also compared our electrophysiological data of N_inf_ cells with those N_inf_ data previously reported [14] and found out that they are very similar.

The present study shows that the concentration-effect relationship for blockade AP of firing between 0.1 until 1000.0 nM melatonin, expressed as percentage of cells, which underwent AP blockade, did not show a clear variation of effect magnitude with concentration variation. The number of neurons in which blockade occurred was in average approximately 35%, ranging from 23.0 to 40.0%. A similar type of concentration-effect relationship was observed for the increase of rheobase in EMS cells. This shows that melatonin blocking effect on N_inf_ neurons, concerning concentration, saturates at 0.1 nM and also shows that the percentage of N_inf_ neurons susceptible to AP blockade is only approximately 35% of that population. It thus suggests that a large percentage of N_inf_ cells are not sensitive to the excitability blocking effect of melatonin. The measurement of rheobase, in the presence of melatonin, found out a remarkable increase of this parameter in all tried concentrations ≥0.1 nM in EMS neurons, which fully agrees with the pattern of percentage of N_inf_ cells with melatonin-induced AP blockade. The alteration of rheobase did not occur in EMNS cells, which shows not only the coherence on the modification of the value of this parameter with the percentage of neurons with AP firing inhibited, but also the clear-cut difference of melatonin effects on EMS and EMNS neurons concerning excitability.

The decrease in neuronal excitability, caused by melatonin, was already described [4,15,19,20]. In N_0_ neurons, it was found that melatonin also blocks neuronal excitability, but with a smaller pharmacological potency and a different pattern of effect-concentration dependency, since blockade occurred at [melatonin] ≥ 10.0 nM and the percentage of blockade increased with concentration [15]. This data set suggest that the EMS N_inf_ cells were greatly more sensitive to melatonin action that the N_0_ cells, since 0.1 nM was already effective to block neuronal excitability on this population [15]. Despite the AP blockade caused by 0.1 nM melatonin in EMS cells, the passive properties were not changed (RMP and R_in_), which shows that in these neuron, pharmacologically, the most potency is on excitability and active related parameters. Additionally, it should be mentioned that previous work already reported the inhibitory effect on excitability, caused by melatonin 10.0 pM on a population of neurons of the central nervous system, highlighting the remarkable potency of this neurohormone [21].

Concerning the passive electrophysiological properties of the neurons, we observed a hyperpolarization of the RMP after melatonin exposure. This hyperpolarization effect, caused by melatonin, was reported in several works [21,22,23]. Although this effect was similar to that previously described [15], the magnitude of the hyperpolarization showed a tendency to be greater in N_inf_ neurons. While in N_0_ neurons, the maximum hyperpolarization was 3.07 mV, in N_inf_ neurons we found hyperpolarization up to 5.77 mV (which correspond to an increase of 10.1%) at melatonin 1.0 nM concentration (Figure 3), which strengthens our hypothesis of a greater sensibility of N_inf_ neurons to melatonin if compared to N_0_.

One explanation to the hyperpolarization of RMP can be associated with changes in membrane conductance to cations current. If melatonin increases the membrane conductance to outward cationic current, e.g., voltage-dependent potassium currents, the results would be a hyperpolarization of neuronal RMP. In accordance with the increase in membrane conductance, a reduction in membrane input resistance would be expected. However, our data showed hyperpolarization of RMP, an increase in input resistance and, consequently, a reduction in membrane conductance. Thus, an alternative attractive hypothesis to explain the RMP hyperpolarization is the melatonin effect on Na^+^K^+^ATPase pump. This protein carries 3 Na^+^ ions to extracellular and 2 K^+^ ions to intracellular medium with a consumption of ATP. So an electrogenic pump is considered that contributes to the electronegativity of the RMP of approximately 5 mV in neurons [24,25]. If melatonin increases the Na^+^K^+^ATPase activity, we could expect an increase in the Na^+^/K^+^ ions exchange and an increase in its contribution to the electronegativity of RMP. In order to investigate this hypothesis, we evaluated the DRG Na^+^K^+^ATPase activity and we found out that this hormone increased the activity of this pump through an acute effect. This result is in agreement with previous reports which described the melatonin ability to increase the activity of this pump in other structures [26,27,28].

Regarding the R_in_, melatonin did not significantly change this parameter in the EMS cells. On the other hand, in EMNS cells, an increase in R_in_ at nanomolar range of concentration, which was statistically different from control at 1.0, 10.0, and 1000.0 nM, was observed. This suggests that the alteration of R_in_ is an effect unrelated to the inhibition of excitability blockade. Additionally, since R_in_ represents a decrease in cell conductance, it is unlikely to be due to alteration of the major RMP determinant: membrane conductance to K^+^ (gK). This is because a decrease in gK would cause membrane depolarization, since K^+^ equilibrium potential is negative to the resting potential [29]. Indeed, our results suggest that melatonin-induced hyperpolarization and increase in R_in_ are effects with independent causal link. This is because, while the increase in R_in_ occurred only in EMNS cells, significant RMP hyperpolarization was observed in both EMS and EMNS neurons, which suggest that these RMP and R_in_ alteration very likely do not hold a causal relationship. The increase in R_in_ was already described by other reports [15,30] and is a conspicuous effect in N_0_ cells of DRG [15].

Melatonin altered several AP parameters in EMS neurons. The parameter most conspicuously altered was the (dV/dt)_max_ of AP depolarization, which showed a tendency for inhibition at all concentrations (0.1–1000.0 nM) and a significant inhibition at 1.0 and 100.0 nM. This is coherent with the melatonin-induced blockade of AP triggering on EMS neurons, since (dV/dt)_max_ is closely related to the inward Na^+^ current, the major cause of the neuronal AP [31]. In coherence with that is also the robust melatonin-induced increase in rheobase in EMS N_inf_ cells, which is a parameter used for fine quantification on the measurement of the neuronal excitability alteration [32]. Still concerning coherence, in EMNS cells melatonin (0.1–1000.0 nM) induced no alteration of (dV/dt)_max_ of AP depolarization, neither of the rheobase, as expected, given the fact that the excitability of these neurons were not altered.

Previous reports have studied neurons showing inflections on the repolarization phase of the AP and with AP duration very similar to N_inf_ neurons [13,14,16,17,18]. Based on the conduction velocity of the axons connected to these neurosomas, these neurons were classified as type C sensory neurons, and, more specifically, as nociceptors. Since only a fraction of N_inf_ cells were sensitive to melatonin, this raises the question whether, from the point of view of physiological functions, these cells would constitute a particular type of nociceptors.

In conclusion, we have here demonstrated that melatonin acts on some N_inf_ cells, the EMS neurons, decreasing their excitability. Comparing our results with those in previous studies, in N_0_ neurons [15], it is concluded that the activity of melatonin on N_inf_ EMS neurons is done with a largely increased pharmacological potency. This inhibition of excitability is done with such a great increase in pharmacological potency so as to suggest that, on some DRG neurons, it exerts important hormonal activity. Besides that, the melatonin effect on excitability, a major neuronal function, performed with the greatest pharmacological potency, is restricted to a sub-set of N_inf_ cells, showing a great relative specificity for those neurons. Taking these facts together and the available information that the N_inf_ cells are more closely related to pain sensation [14,33], it seems that an attractive hypothesis is that melatonin acts on DRG on the modulation of specific type of pain sensation. Should this hypothesis be correct, which demonstration remains to be done, melatonin may turn also to be a potentially important therapeutic agent.

## 4. Materials and Methods

### 4.1. Animals and Tissue Dissection

Male Wistar rats (200–300 g) were obtained from the Superior Institute of Biomedical Sciences, State University of Ceará. The animals were kept under a 12 h:12 h light/dark cycle with food and water available ad libitum. All procedures were approved by the animal Ethics Committe of State University of Ceará (CEUA-UECE process: 12777097-6, approved in August 2013). All animals used were sacrificed at Zeitgeber Time (ZT) 3 (3 h after lights on).

Rats were sacrificed by CO_2_ inhalation, DRG’s were dissected from lumbar segments L4 and L5 and immediately placed in modified Locke’s solution. For intracellular recordings, intact tissues were used on the same day of dissection. All electrophysiological experiments were performed at room temperature (22–26 °C).

### 4.2. Solutions and Drugs

The modified Locke’s solution was used for tissue nutrition, whose composition was: 140 mM NaCl, 5.6 mM KCl, 1.2 mM MgCl_2_, 2.2 mM CaCl_2_, 10 mM glucose, and 10 mM Tris-(hydroxymethyl-aminomethane). The pH of Locke’s solution was adjusted to 7.40 with HCl and NaOH.

Melatonin was dissolved in absolute ethanol and stock solutions were prepared daily. The final concentration of ethanol never exceeded 0.04% (*v*/*v*). Stock solutions were added to modified Locke’s solution for electrophysiological recordings. Melatonin concentrations, used for intracellular recordings were 0.01, 0.1, 1.0, 10.0, 100.0, and 1000.0 nM. All salts and drugs were obtained from Sigma Chemical (St. Louis, MO, USA) and were of analytical grade.

### 4.3. Electrophysiological Measurements and Analysis

The recording of DRG neurons’ electrical properties was conducted as previously described [14,34,35]. The dissected DRG was immediately placed in Locke solution and fixed on an acrylic chamber with Sylgard 184^®^ at bottom. The superfusion was maintained by a gravity flux and adjusted to 1.0–1.5 mL/min. The chamber was placed on a stereomicroscope (model College Stereo, MLW Intermed, Schöneiche, Germany) and the microelectrode movement and impalement were performed with an electric micromanipulator (model MS 314—Märzhäuser Wetzlar, Wetzlar, Hesse, Germany). Reservoirs containing modified Locke, and melatonin solutions were connected to the chamber by three-way valves that could rapidly switched between the main reservoir and test solutions. After impalement, the electrophysiological recordings were made after 3–5 min to allow the stabilization of neuronal membrane properties. Neurons were exposed for up to 5 min to a given melatonin concentration or less, until it blocked AP, if blockade occurred first. Subsequently, a washout period began by switching to drug-free solution.

Intracellular recordings were made with thin-walled borosilicate glass microelectrodes (1.0 mm OD, 0.6 mm ID, Sutter Instrument., Novato, CA, USA) filled with a 3.0 M KCl solution. The microelectrodes were pulled with a micropipette puller (P-97 micropipette puller model, Sutter Instruments, Novato, CA, USA) and had resistance ranging from 40 to 90 MΩ. Micropipettes were connected via an Ag–AgCl wire to an Axoclamp 900 A amplifier (Axon Instruments, San Jose, CA, USA).

Neurons were considered to be acceptable for study when they were stabilized with a resting membrane potential, more negative than −45 mV and had an overshoot. APs were elicited in response to depolarizing current pulses, which were 25% above the AP threshold to avoid small variations in experimental conditions that could affect analysis. Current and voltage outputs were sampled at 50 kHz, and data acquisition and storage were performed using computer acquisition hardware (Digidata 1440A model, Molecular Devices, San Jose, CA, USA). A frequency of 1 Hz was used to stimulate preparation and record APs in melatonin and drug-free solutions. The software used for data acquisition was Pclamp 10.4 (Molecular Devices).

The electrophysiological measurements were RMP, R_in_, rheobase and AP parameters: Amplitude, duration, maximum rate of rise [ascendant, dV/dt(asc)], and maximum rate of fall of AP [descendant inclinations, dV/dt(desc); unless otherwise stated, the absolute value of the minimum value of negative dV/dt]. RMP is the resting membrane potential. R_in_ is the membrane input resistance and it was measured by means of Ohm’s law (dividing the maximum voltage response to hyperpolarizing small current pulse). For an accurate measurement of the R_in_, especially when small changes in electrode resistance occur during an experiment, bridge and discontinuous current clamp (DCC) modes were used. In the bridge mode the error in R_in_ measurements depends on incorrect microelectrode resistance compensation, whilst in DCC, it depends on sampling frequency and membrane capacitance and not on microelectrode resistance. Thus, since Bridge and DCC modes have two different sources of error, the agreement of the results collected through the use of the two methods should be considered accurate, and this was used here [36]. The AP amplitude was measured by the difference between maximum voltage amplitude in an AP and RMP. AP duration was measured at half-width maximum amplitude.

### 4.4. Determination of Na^+^/K^+^-ATPase Activity

Na^+^/K^+^-ATPase activity was assayed based on previous works [37,38]. Briefly, a pool of 6 DRGs, dissected from lumbar segments L4 and L5, previously carefully clean of exceeding connective tissues, were homogenized in a solution containing: 250 mM sucrose, 10 mM HEPES-Tris buffer, 2 mM ethylenediamine tetraacetic acid (EDTA), and 1 mM phenylmethylsulfonyl fluoride (PMSF). The homogenization was made with a tissue homogenizer (FISATOM 713D model) and the homogenate was kept on ice during the process.

Afterwards, protein concentration measurements were done according to the method of Lowri et al. [39]. The homogenate was separated in control (vehicle) and experimental (melatonin) groups. In the experimental group, the homogenate was pre-incubated with melatonin (100.0 nM) added to the assay mixture (composition: 120 mM NaCl, 30 mM KCl, 4 mM MgCl_2_, 20 mM HEPES-Tris, and 5 mM ATP-Na^+^) for 15 min at 37 °C. The control group was exposed only to the assay mixture. After the pre-incubation period, it was added ouabain to the homogenates and last 45 min at 37 °C. At the end of this period, the reaction was stopped by the addition of trichloroacetic acid and standard curves were made to quantify Na^+^/K^+^-ATPase pump activity.

Ouabain-sensitive Na^+^/K^+^-ATPase activity was quantified by the difference between total ATPase and ouabain-insensitive activity and was expressed as nmol of inorganic phosphate (Pi) released from ATP per µg protein per hour at 37 °C. The amount of Pi released was quantified colorimetrically, as previously described [40] using KH_2_PO_4_ as reference. The values obtained were measured in triplicates.

### 4.5. Statistical Analysis

Data are reported as means ± SEM with “*n*” indicating the numbers of experiments. The experimental groups were normalized by its own internal control in each experiment, for each melatonin concentration. The paired Student’s *t*-test was used to compare a pair of values (control and experimental) of raw data (non-normalized) collected on the same preparation. Data were considered to be significant when *p* < 0.05. Comparisons of data collected on different preparation, parametric ANOVA followed by Holm-Sidak post hoc test was used.

## Figures and Tables

**Table 1:** Electrophysiological parameters of N_inf_ (excitability melatonin sensitive (EMS) and excitability melatonin not sensitive (EMNS)) and N_0_ neurons in control situation.

Parameters ^1^	N_inf_ Parameters (EMS) ^5^	N_inf_ Parameters (EMNS) ^6^	N_0_ Parameters ^7^
RMP ^2^ (mV)	−56.0 ± 0.98 ^#^	−59.3 ± 0.93	−58.8 ± 0.59
Input resistance (MΩ)	31.8 ± 9.22	63.6 ± 14.69 *	13.0 ± 0.93
Rheobase (nA)	1.5 ± 0.14	1.6 ± 0.10	1.5 ± 0.07
Amplitude (mV)	80.8 ± 2.13 *	78.6 ± 1.34 *	71.3 ± 0.88
Duration (ms)	1.9 ± 0.16 *	2.1 ± 0.19 *	0.8 ± 0.02
dV/dt_asc_^3^ (V/s)	115.7 ± 5.85 *	110.6 ± 5.47 *	167.1 ± 4.81
dV/dt_desc_^4^ (V/s)	−54.1 ± 2.69 *	−58.4 ± 2.56 *	−108.5 ± 3.14

^1^ Data are expressed as means ± SEM. ^2^ RMP: Resting membrane potential. ^3^ dV/dt_asc_: Maximum ascendant inclination. ^4^ dV/dt_desc_: Maximum descendant inclination. ^5^ EMS: excitability melatonin sensitive. ^6^ EMNS: excitability melatonin not sensitive. ^7^ Data from previous published study: [15]. The symbol * indicates statistical difference compared to N_0_ neurons parameters (*p* < 0.05, ANOVA, followed by Holm-Sidak test). The symbol ^#^ indicates statistical difference compared to EMNS neurons parameters (*p* < 0.05, ANOVA, followed by Holm-Sidak test). EMS did not differ from EMNS cells, except for the RMP.

## Abbreviations

AP	Action potential
DRG	Dorsal root ganglia
dV/dt_asc_	Maximum ascendant inclination
dV/dt_desc_	Maximum descendant inclination
EMNS	Excitability melatonin not sensitive
EMS	Excitability melatonin sensitive
R_in_	Input resistance
RMP	Resting membrane potential
SCN	Suprachiasmatic nucleus
